# A normalized parameter for comparison of biofilm dispersants *in vitro*

**DOI:** 10.1016/j.bioflm.2024.100188

**Published:** 2024-03-03

**Authors:** Shuang Tian, Linqi Shi, Yijin Ren, Henny C. van der Mei, Henk J. Busscher

**Affiliations:** a University of Groningen and University Medical Center Groningen, Department of Biomedical Engineering, Antonius Deusinglaan 1, 9713, AV, Groningen, the Netherlands; b State Key Laboratory of Medicinal Chemical Biology, Key Laboratory of Functional polymer Materials of Ministry of Education, Institute of Polymer Chemistry, College of Chemistry, Nankai University, Tianjin, 300071, PR China; c University of Groningen and University Medical Center Groningen, Department of Orthodontics, Hanzeplein 1, 9700, RB, Groningen, the Netherlands

**Keywords:** Dispersed bacteria, Extracellular polymeric substances, eDNA, Micelles, Sepsis

## Abstract

Dispersal of infectious biofilms increases bacterial concentrations in blood. To prevent sepsis, the strength of a dispersant should be limited to allow the immune system to remove dispersed bacteria from blood, preferably without antibiotic administration. Biofilm bacteria are held together by extracellular polymeric substances that can be degraded by dispersants. Currently, comparison of the strength of dispersants is not possible by lack of a suitable comparison parameter. Here, a biofilm dispersal parameter is proposed that accounts for differences in initial biofilm properties, dispersant concentration and exposure time by using PBS as a control and normalizing outcomes with respect to concentration and time. The parameter yielded near-identical values based on dispersant-induced reductions in biomass or biofilm colony-forming-units and appeared strain-dependent across pathogens. The parameter as proposed is largely independent of experimental methods and conditions and suitable for comparing different dispersants with respect to different causative strains in particular types of infection.

## Highlights

• A parameter is proposed for comparing the strength of biofilm dispersants.
• The parameter accounts for differences amongst studies in dispersant concentration.
• The parameter accounts for differences amongst studies in exposure time.
• The parameter is independent of the method used to quantitate biofilm dispersal.
• The parameter differs for biofilms of different bacterial pathogens.

## 1 Introduction

Bacteria in an infectious biofilm are glued together by their self-produced matrix of extracellular polymeric substances (EPS) [1]. Main components of the EPS matrix are eDNA, proteins and polysaccharides [2]. In an infection control strategy based on dispersal of biofilm bacteria, each of these main components of the EPS matrix can be the target of degradation by a dispersant [3,4]. The potential success of biofilm dispersants as a clinically applicable strategy to control infectious biofilms, critically depends on an adequate host immune response to the sudden increase in the concentration of dispersed bacteria in blood [5,6]. Metaphorically, Wille and Coenye compared biofilm dispersal strategies with the opening of Pandora's box [7], in which Pandora's box represents the biofilm in which bacterial pathogens are safely tucked away. Upon opening the box, disasters are released and disperse over the world, i.e. upon biofilm dispersal the dispersed pathogens may cause life-threatening sepsis. Dispersal of motile, infectious bacteria from infected wounds using glycoside hydrolases for instance, caused lethal sepsis in mice when not used in combination with a suitable antibiotic therapy [8]. However, considering the rise in the number of antibiotic-resistant bacteria [9], dispersants yielding a balanced increase in the concentration of dispersed bacteria in blood that can be handled by the host immune system without the need for additional antibiotic therapy are preferable.

A large number of different synthetic biofilm dispersants as an alternative for antibiotics in the treatment of bacterial infection has been forwarded in recent literature, evaluated using different experimental models, methods and conditions. As a result, comparison of the strength of dispersants developed in different studies based on the evaluation of different outcomes [6,8,[10], [11], [12], [13], [14], [15], [16]] is impossible by lack of a suitable comparison parameter. *In vivo* methods to obtain the required outcomes for calculation of a comparative parameter are scarce and need inclusion of a large number of animals. Therefore, such an parameter would necessarily have to be based on comparison of the dispersal of biofilms *in vitro* and account for differences in initial biofilm properties, exposure time and dispersant concentrations, representing the major experimental variables in different experimental models. Importantly, well-designed *in vitro* experiments evaluated with an appropriate parameter may reduce the need for animal experiments. Most methods to evaluate biofilm dispersal are based on qualitative micrographs, or more quantitative outcomes such as reductions in biomass, biofilm thickness or the number of colony forming units (CFUs) in a biofilm [17]. However, neither qualitative images nor quantitative outcomes of an assay can be directly used for quantitative comparison without accounting for initial differences in biofilm properties, exposure time to dispersants and dispersant concentration. This impedes a simple comparison of different dispersants developed and evaluated in different experimental models. Yet, with the increasing interest in biofilm dispersal as an infection control strategy and the accompanying increase in the number of dispersants forwarded in the literature, the need for a quantitative biofilm dispersal parameter applicable to different experimental models, methods and conditions, is increasing as well.

The aim of this article is to propose an *in vitro* biofilm dispersal parameter (BDP) that accounts for differences in initial biofilm properties, exposure time and concentration of dispersants applied. The BDP proposed is validated here based either on a dispersant-induced reduction in biomass or CFUs in biofilm remaining after exposure to a dispersant, but may also be based on evaluation of other dispersant-induced changes in biofilm characteristics. As an example of its use and without aiming to go into a detailed analysis of the mechanisms of micellar dispersants, the parameter proposed is employed here for a quantitative comparison of different micellar dispersal systems (Table 1).

**Table 1:** Stimuli-responsive, micellar dispersants used in this study, their composing polymers, drug loading content (DLC) by the active dispersant component and biofilm matrix components to be degraded.

Abbreviation	Polymeric components^a^	DLC of active dispersants (%)	Matrix targets	Ref.
PEG/PQAE	PEG-*b*-PCL/PCL-*b*-PQAE	PQAE (100^b^)	eDNA, amyloid fibers	[13]
PEG/PAE-EGCG	PEG-*b*-PCL/PCL-*b*-PAE	EGCG (23)	Amyloid fibers	[14]
PEG/PAE-DNase	PEG-*b*-PCL/PCL-*b*-PAE	DNase I (8)	eDNA	[15]

PCL-*b*-PQAE, PCL-*b*-poly(quaternary amino ester); PCL-*b*-PAE, PCL-*b*-poly(amino ester); EGCG, (−)-Epigallocatechin gallate.

a Abbreviations: PEG-*b*-PCL, Poly (ethylene glycol)-*b*-poly (ε-caprolactone).

b Implying that micelles themselves are the active dispersants, without any additional loading.

## 2 Materials and methods

### 2.1 Micellar dispersants used

The micellar dispersants summarized in Table 1 were all prepared as previously reported [[13], [14], [15]] without any additional antimicrobial core-loading. In essence, all micelles were prepared through self-assembly of the composing surfactants in a polar solvent (dimethyl sulfoxide) and have been demonstrated to be stimuli-responsive (i.e. pH-responsive) and accumulate in an infectious biofilm upon tail-vein injection in mice. Zwitterionic PEG/PQAE micelles were used as a dispersant and shown to interact with eDNA and amyloid proteinaceous fibers. EGCG was cross-linked with PAE through pH-reversible boronic-ester binding, allowing release of EGCG inside an infectious biofilm to disassemble amyloid protein fibers. DNase I in PEG/PAE micelles was conjugated to PAE to become part of the micellar shell where it was shown to be protected against inactivation by blood-borne enzymes. In the acidic environment of an infectious biofilm, PAE stretches to expose the conjugated DNase I and allows it to become active in degrading eDNA. EGCG was also included in the shell of PEG/PAE micelles in order to be protected against inactivation at physiological pH.

### 2.2 Bacterial culturing and harvesting

*Enterococcus faecium* ATCC 35667, *Staphylococcus aureus* ATCC 12600, *Klebsiella pneumoniae*-1, *Acinetobacter baumannii* ATCC 19606, *Pseudomonas aeruginosa* PAO1 and *Enterobacter cloacae* BS 1037 were grown from frozen stock on blood agar plates at 37 °C for 24 h. For pre-cultures, a single bacterial colony was transferred into 10 mL of tryptone soy broth (TSB, OXOID, Basingstoke, UK) and incubated 24 h at 37 °C. For main cultures, the pre-culture was transferred into 200 mL of TSB and incubated for 16 h at 37 °C. Then, bacteria were harvested by centrifugation (5000 g, 5 min, 10 °C) followed by washing twice in sterile phosphate buffered saline (PBS, 5 mM K_2_HPO_4_, 5 mM KH_2_PO_4_, and 150 mM NaCl, pH 7.0). The bacterial suspension was mildly sonicated three times each for 10 s with 30 s intervals between each cycle on ice to obtain a suspension with single bacteria for initiating bacterial adhesion and biofilm formation. Absence of lysis was microscopically (phase-contrast) established during measurement of bacterial concentrations in suspension using a Bürker–Türk counting chamber. After counting, the final bacterial concentration was fixed at 1 × 10^9^ bacteria/mL for further experiments. Calibration of total chamber counts *versus* viable bacteria culturable on agar plates demonstrated ≫ 95% viability.

### 2.3 Biofilm formation

For biofilm formation, bacterial suspensions in PBS (0.5 mL) were put into sterile polystyrene 48-well plates for 2 h at room temperature to allow bacterial adhesion. Next, the suspensions were removed, wells were washed three times with sterile PBS, filled with fresh TSB (1 mL) and incubated for 48 h at 37 °C under static conditions to prevent detachment of bacteria from the well surfaces. The resulting biofilm was exposed to a crystal violet solution (1%, w/v) which was added to each well. After 20 min, the crystal violet solution was removed and wells were gently rinsed three times with PBS to remove remaining crystal violet solution. After rinsing, 500 μL of 33% acetic acid was added to resuspend stained biofilms for 15 min and absorbance in each well was read on a microplate reader (Thermo Fisher Scientific Inc., Waltham, USA) at 595 nm as a measure for the amount of biomass in a biofilm. In case the OD was higher than 1, the crystal violet solution was diluted and the absorbances measured were multiplied with the dilution factor for the calculation of the amount of biomass. Alternatively, the biofilm after rinsing with PBS was removed by scraping and bacteria were suspended in PBS. Subsequently, the resulting suspension was mildly sonicated to break bacterial aggregates, serially diluted, plated on TSB agar and incubated at 37 °C. After 24 h, the number of CFUs in the biofilm was enumerated and expressed in CFU (log-units) per cm^2^.

### 2.4 Biofilm dispersal

For measuring biofilm dispersal, biofilms grown as described above were first washed with PBS to remove TSB and subsequently exposed to 0.5 mL of a micellar suspension (200 μg/mL) in PBS for 120 min. Biofilms in PBS without exposure to micelles were taken as a control, which not only accounts for differences in initial biofilm thickness but also for differences in other biofilm properties of possible influence. Note, that micellar concentrations can be easily transformed into dispersant concentrations based on the loading content of the micelles (see Table 1). After exposure, the suspension above the biofilm was removed and biomass and CFUs were determined as described above in section 2.3.

Since *S. aureus* counts as one of the most frequently occurring human pathogens, the dispersal of *S. aureus* was visualized as an example of dispersal events at a microscopic level. To this end, *S. aureus* ATCC 12600 biofilms, grown on glass slides placed on the bottom of 48-well plates and exposed to dispersants as described above, were imaged using scanning electron microscopy (SEM). After exposure to a dispersant, glass slides with adherent biofilms were fixed with 2.5% glutaraldehyde for 2 h at 4 °C, removed from the wells, dehydrated with a series of ethanol solutions and gold sprayed for SEM (Quanta 200, FEI, Hillsboro, USA) at an acceleration voltage of 15 kV.

### 2.5 Statistical analysis

All experiments were done with three, separately grown bacterial cultures, representing biological replicates. Occasionally, technical replicates were taken, yielding near-identical results. Therefore, only standard deviations over biological replicates were reported and differences between two groups were examined for statistical significance based on biological replicates, using a two-tailed, paired Student's *t*-test. Multivariate parametric data were analyzed using analysis of variance (ANOVA) with Tukey's post hoc test. Statistical significance between groups was accepted at *P* < 0.05.

## 3 Results

### 3.1 Initial biofilm formation

**Fig. 1:**
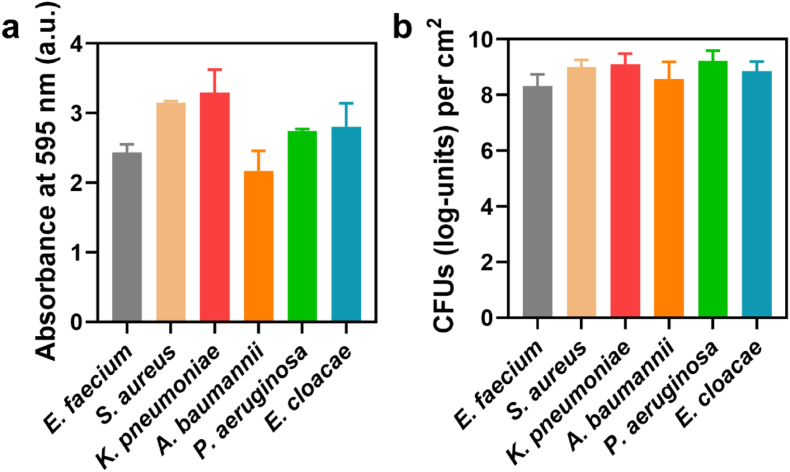
**Initial biofilm formation by different ESKAPE-panel pathogens in TSB after 48 h growth at 37°C. After growth, samples were washed with PBS to remove remaining growth medium. (a)** Absorbances (595 nm) of biofilms after crystal violet staining, as a measure for the biomass of biofilms. **(b)** The number of CFUs (log-units) retrieved per cm^2^ substratum surface from biofilms determined by plate counting. All error bars denote standard deviations over three experiments with separately grown bacterial cultures. (For interpretation of the references to colour in this figure legend, the reader is referred to the Web version of this article.)

Biofilms of ESKAPE-panel, i.e. bacterial pathogens [18] that are able to escape current antibiotic treatment, were grown in TSB at 37 °C for 48 h. After growth, biofilms were either stained with crystal violet and absorbances measured (Fig. 1a) or the number of CFUs were enumerated after scraping off the biofilm and agar plating (Fig. 1b). All bacterial strains were capable of forming biofilms with high CFU counts. Both initial biomass, as derived from absorbance measurements as well as the initial number of CFUs (log-units) retrieved per cm^2^ varied across the different ESKAPE-panel pathogens, emphasizing the need to use PBS as a control to compensate for differences in initial biofilm properties in the definition of a Biofilm Dispersal Parameter.

### 3.2 Determination and definition of the biofilm dispersal parameter

In a separate experiment, biofilms were exposed for a standardized period of time (120 min) to a micellar suspension (200 μg/mL). In order to create a generally applicable Biofilm Dispersal Parameter (BDP) that can be employed for comparison with other studies in which different exposure times and dispersant concentrations are used, biofilm dispersal must be normalized with respect to time and dispersant concentration. For normalization, we here propose to express exposure time in minutes and normalize to an exposure time of 1 min. For dispersal concentrations, we propose to normalize to a dispersant equivalent concentration of 1 μg/mL. Dispersant equivalent concentrations can be calculated based on the loading content of a dispersant in a micellar carrier. Accordingly, assuming a linear increase in outcome of an assay with time and concentration, a normalized change in outcomes after exposure to dispersants, can be calculated according to

(1) $$Normalized\;Outcome\;Change=\left(Outcome\;\left(control\right)-Outcome\;\left(dispersant\right)\right)\times \frac{1}{exposure\;time}\times \frac{1}{concentration\;applied}$$

in which *Outcome (Control)* is the experimental outcome for a biofilm that has not been exposed to a dispersant but solely to a PBS *control* and *Outcome (dispersant)* is the experimental outcome for a biofilm that has been exposed to a dispersant for a specified *exposure time* (to be expressed in min) and *concentration applied* (to be expressed in μg/mL). Note, that in order to make the BDP a dimensionless number, 1 min and 1 μg/mL have been chosen as a standard for normalizing exposure time and dispersant concentration. Expressing of the outcome relative to a PBS control accounts for possible differences in initial biofilm thickness. Normalization with respect to exposure time and dispersant concentration allows to compare results from studies applying different exposure times and dispersant concentrations.

Subsequently, a BDP can be calculated either using biomass obtained from absorbance after crystal violet staining (BDP_CV_) or using the number of CFU (BDP_CFU_)

(2) $$\begin{array}{c} BDP=\frac{Normalized\;Outcome\;Change}{Outcome\;\left(control\right)\;} \end{array}$$

A resulting BDP equal to 0 represents absence of biofilm dispersal.

Thus calculated BDPs are summarized in Table 2 for biofilms grown from different ESKAPE-panel strains, exposed to each of the three micellar dispersants in Table 1. As an important conclusion from Table 2, BDPs derived from biomass and CFU enumeration are similar (*P* > 0.05, two-tailed, paired Student's *t*-test). PEG/PQAE micelles yielded the lowest BDP across all six ESKAPE pathogens, while PEG/PAE-DNase micelles yielded the highest BDP. Interestingly, PEG/PAE-EGCG micelles previously suggested to yield a balanced dispersal that could be handled by the host immune system [14], had intermediate BDP values. PEG/PAE-DNase micelles yielded most dispersal of *S. aureus*, *K. pneumoniae*, *A. baumannii* and *P. aeruginosa* biofilms, while performing least against biofilms of *E. faecium* and *E. cloacae*.

**Table 2:** **Biofilm dispersal parameters (BDPs) of ESKAPE-panel pathogens by different micellar dispersants** (see Table 1 for details). Data represent averages with standard deviations over three separate bacterial cultures and separately prepared micelles.

Strains	BDP_CV_ × 10^5^			BDP_CFU_ × 10^5^		
	PEG/PQAE	PEG/PAE-EGCG	PEG/PAE-DNase	PEG/PQAE	PEG/PAE-EGCG	PEG/PAE-DNase
*E. faecium*	1.3 ± 0.5	6.9 ± 0.4	8.3 ± 0.4	1.4 ± 0.5	6.4 ± 0.4	10.5 ± 1.3
*S. aureus*	3.1 ± 0.5	8.0 ± 0.4	24.9 ± 1.6	3.0 ± 0.5	7.8 ± 0.4	24.3 ± 0.8
*K. pneumoniae*	2.4 ± 0.5	5.9 ± 0.4	21.3 ± 0.4	2.5 ± 0.5	5.5 ± 0.4	22.2 ± 0.8
*A. baumannii*	2.6 ± 0.5	8.3 ± 0.4	23.5 ± 0.8	2.8 ± 0.5	7.7 ± 0.4	22.6 ± 1.7
*P. aeruginosa*	3.1 ± 0.5	9.2 ± 0.4	24.3 ± 0.8	3.3 ± 0.5	8.7 ± 0.4	22.2 ± 0.4
*E. cloacae*	1.7 ± 0.5	4.9 ± 0.4	11.9 ± 0.4	2.1 ± 0.5	5.4 ± 0.4	11.3 ± 0.4

**Fig. 2:**
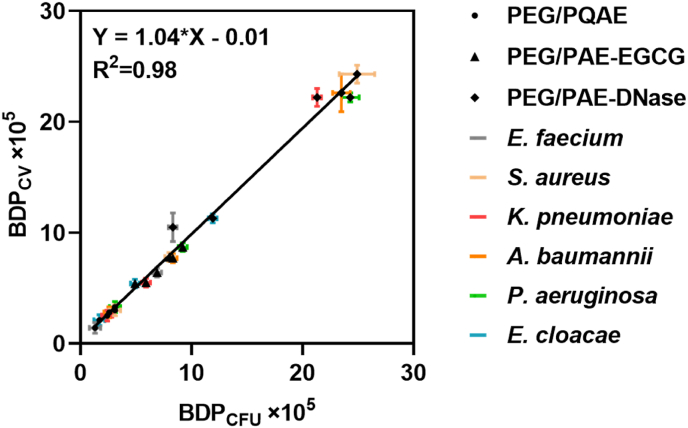
**BDP**_**CV**_**as a function of the BDP**_**CFU**_**for three different micellar dispersants as evaluated for six different ESKAPE-panel pathogens.** Data (see Table 2) represent averages with standard deviations over three separate bacterial cultures and separately prepared micelles.

In a graph of BDP_CV_ *versus* BDP_CFU_, data spread on the line of identity (Fig. 2) with a high linear correlation coefficient of 0.98, suggesting independence of the method employed to evaluate dispersal using the parameter proposed.

### 3.3 Biofilm dispersal parameter versus microscopic dispersal events visualized by SEM

**Fig. 3:**
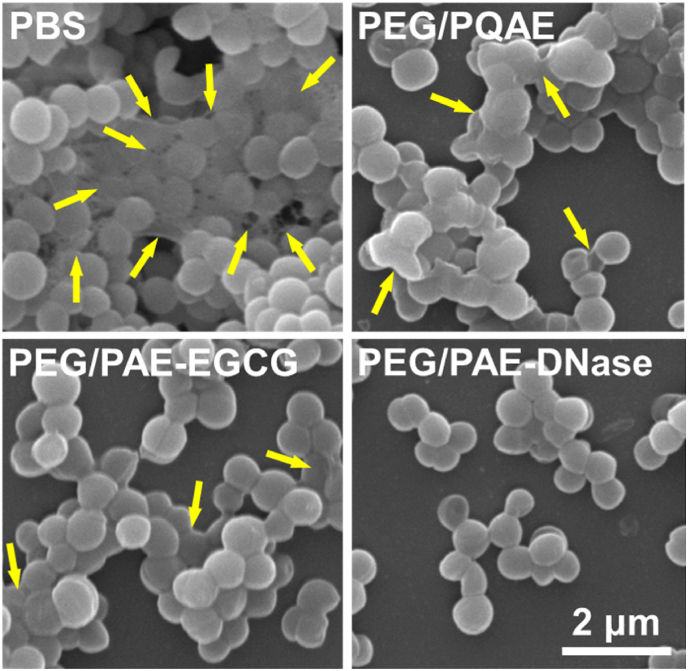
**SEM micrographs of *S. aureus* biofilms grown under the same conditions after exposure to PBS as a negative control or different micellar dispersant systems** (see Table 1 for details). Note abundant EPS in biofilms exposed to PBS, and absence of large bacterial aggregates after exposure to PEG/PAE-DNase. Yellow arrows indicate EPS. (For interpretation of the references to colour in this figure legend, the reader is referred to the Web version of this article.)

Imaging of *S. aureus* biofilms prior to dispersal using SEM (Fig. 3) showed a dense biofilm comprised of large aggregates that are all connected with visible EPS after exposure to PBS. Dispersal by exposure to the different micellar dispersants becomes evident from absence of visible EPS and increasing occurrence of small aggregates and less dense biofilm with more open structure. Biofilm density as judged from these images, decreased from exposure to PBS as the negative control (BDP = 0), to PEG/PQAE (BDP = 3.1 × 10^−5^), PEG/PAE-EGCG (BDP = 8.0 × 10^−5^) to PEG/PAE-DNase micelles (BDP = 24.9 × 10^−5^), which constitutes a similar ranking as can be obtained based on our proposed biofilm dispersal parameter and therewith constitutes a microscopic validation of the BDP proposed.

## 4 Discussion

We propose a quantitative biofilm dispersal parameter (BDP) for the comparison of biofilm dispersants that can be applied to compare the strength of different dispersants *in vitro*. BDP values correspond with microscopic visualization of dispersal event and are compensated for differences in initial biofilm thickness prior to exposure to a dispersant by expressing itself relative to PBS as a control. Also, the definition of the BDP proposed accounts for the use of different concentrations and exposure times applied by normalization of the data to an exposure time of 1 min and a dispersant equivalent concentration of 1 μg/mL. This normalization is based on the assumption that dispersal increases linearly with exposure time and concentration [14,19,20]. For *in vitro* evaluation, dispersal usually follows an exponential increase that is initially linear and levels off after prolonged exposure times. Similarly, overly high concentrations above which dispersal levels off, are clinically useless and even dangerous leading to blood levels of dispersed bacteria that the immune system cannot deal with [6], are sometimes not biosafe [8] and overly expensive [21].

As presented here, the parameter requires biomass or CFUs of two or more biofilms grown under the same conditions treated with a dispersant and a PBS control. BDP_CV_ and BDP_CFU_ values obtained for the same dispersant and the same bacterial strain were statistically identical (Table 2) when derived from biomasses obtained using crystal violet staining or from CFU enumeration of biofilms after agar plating as assay outcomes. Moreover, in a graph of BDP_CV_ *versus* BDP_CFU_, data spread on the line of identity (Fig. 2). Accordingly, the BDP determined for a particular bacterial strain and dispersant is identical whether derived from crystal violet staining or from CFU enumeration. It is within reason to anticipate that application of Eqs. (1), (2)) also yields identical BDP values when using outcomes from other types of assays.

This is different from the conclusion of a meta-analysis of published data on antimicrobial efficacy of biocides in which the choice of a particular method was mentioned to be the most decisive factor for determining the outcome of an assay [22]. Unlike biocides, dispersants are not aimed at bacterial killing, that is notoriously hard to establish inequivocally, since a) live-bacteria can be non-culturable, b) cell wall damaged bacteria as determined by so-called live-dead assays are often considered death but can be cultured and c) dormant bacteria with a metabolic activity below detection can still be re-vive [[23], [24], [25]]. Dispersal on the other hand, is solely based on the detachment of bacteria from a biofilm, either by sloughing off of large sections of a biofilm and/or erosion of individual bacteria [5]. Thus, as compared with bacterial killing, dispersal can be considered easier to establish more independently of the method applied (Fig. 2).

It is instructive to apply the parameter as proposed to literature data in order to illustrate its value for drawing conclusions from different studies, although this is always within the limitation of possible model differences other than the differences accounted for in the parameter proposed here. Using literature data, BDP values of Dispersin B were calculated based on optical density measurements and found to range from 4.2 × 10^−5^ to 5.0 × 10^−5^ for a mutant devoid of the dspB gene [10]. Amongst a collection of glycoside hydrolases as potential dispersants, α-amylase was calculated based on CFU enumeration to possess a BDP of around 0.8 × 10^−5^ for *P. aeruginosa* and 1.3 × 10^−5^ for *S. aureus*, while BDP values of cellulase were lower (0.4 × 10^−5^) for both *P. aeruginosa* and *S. aureus* [8]. Although within the range of BDP values presented here for micellar dispersants, micellar dispersants generally express higher BDP values, likely because the smart micellar encapsulation applied (see Table 1) is pH responsive. Due to the generally acidic pH in a biofilm, smart, micellar nanocarriers become positively charged inside a biofilm, housing mostly negatively charged bacteria. Consequently, electrostatic double-layer attraction allows positively charged micelles to penetrate more deeply and accumulate in higher concentrations in a biofilm than drugs carried free in solution [26], with a possible impact on dispersal.

The biofilm matrix is composed of many EPS components [2], the production of which is not only strain dependent but also dependent on environmental conditions, including nutrient availability. EPS components produced by all ESKAPE-panel pathogens are summarized in Table 3. PEG/PQAE micelles perform poorly across all ESKAPE strains regardless of their matrix composition, probably because breaking biofilm integrity through electrostatic interactions within the EPS matrix is slower than enzymatic degradation of DNase or chemical disassembly by EGCG. Proteinaceous amyloid or curli fibers occur in all ESKAPE strains together with other matrix components. This explains why PEG/PAE-EGCG micelles that are especially able to disassemble proteinaceous structures perform better than PEG/PQAE micelles across all ESKAPE-panel strains. eDNA is a relatively long molecule, making it a glue that can act over relatively long distances [27]. It occurs abundantly in all ESKAPE biofilms with the exception of *E. faecium* [28] and *E. cloacae* [29]. Accordingly, PEG/PAE-DNase micelles have high BDP values across all ESKAPE strains with the exception of *E. faecium* and *E. cloacae*. Another possible reason of PEG/PAE-DNase failing to disperse *E. faecium* and *E. cloacae* biofilms is that eDNA exists as a complex bound to other EPS components as e.g. alginate and curli fimbriae during biofilm maturation, making it more difficult to be degraded [30,31].

**Table 3:** Major EPS components in biofilms of ESKAPE-panel pathogens.

Strain	EPS component	Growth medium	Ref.
*E. faecium*	eDNA	TSB with glucose	[32]
	Secreted antigen A, Surface protein Esp	TSB with glucose, BHI, TSB	[32,33]
	Alginate	MHB, BHI	[28]
*S. aureus*	eDNA	TSB with glucose	[34]
	PSM amyloid fibers, Surface proteins	BHI, TSB with glucose	[35,36]
	PNAG	TSB with yeast extract and glucose	[19]
*K. pneumoniae*	eDNA	LB	[37]
	Proteins, amyloid fibers	LB	[37,38]
	PNAG	LB with glucose	[39]
*A. baumannii*	eDNA	LB	[40]
	Bap, CSu pili	TSB, LB	[41,42]
	PNAG, lipo-oligosaccharide, capsular polysaccharides	TSB	[43]
*P. aeruginosa*	eDNA	LB, minimal glucose medium	[44,45]
	Matrix-associated protein, FapC amyloid fibers	M9 broth, LB, MHB	[[46], [47], [48]]
	Psl, Pel, alginate	LB	[49,50]
*E. cloacae*	eDNA	TSB	[51,52]
	Curli fimbriae	TSB	[29,51]
	Polysaccharide, cellulose	LB	[53]

Abbreviations: MHB, Mueller Hinton broth; BHI, brain heart infusion; TSB, tryptone soy broth; LB, Luria Bertani broth; PSM, phenol soluble modulin; PNAG, Poly-*N*-acetylglucosamine; Bap, Biofilm-associated protein.

In summary, the biofilm dispersal parameter proposed accounts for differences in initial biofilm thickness, dispersant concentration and exposure time and has been measured for six different members of the ESKAPE-panel of bacterial pathogens. Validation has been done using micellar dispersants, but it is within reason to anticipate that the parameter is also applicable to other dispersant systems. The biofilm dispersal parameter proposed here can assist to determine how far Pandora's box can be safely opened by different dispersants, based on *in vitro* comparisons. As more research groups would adopt the habit of calculating the biofilm dispersal parameter as proposed and connect the values with the *in vivo* performance of the dispersant studied, the parameter will gain more value in relation with predicting septic complications of dispersal and reduce the need for animal experiments.

## Funding

This work was financially supported by the National Key R&D Program of China (project number: 2022YFA1205700), 10.13039/100014717National Natural Science Foundation of China (grant numbers: 51933006 and 52293383) and UMCG.

## CRediT authorship contribution statement

**Shuang Tian:** Writing – original draft, Methodology, Investigation, Formal analysis, Data curation, Conceptualization. **Linqi Shi:** Writing – review & editing, Supervision, Conceptualization. **Yijin Ren:** Writing – review & editing. **Henny C. van der Mei:** Writing – review & editing, Supervision, Investigation, Data curation, Conceptualization. **Henk J. Busscher:** Writing – review & editing, Supervision, Formal analysis, Data curation, Conceptualization.

## Declaration of competing interest

H.J.B. is the director-owner of a consulting company, SASA BV. The authors declare that they have no potential competing interests with respect to authorship and/or publication of this article.

## Data Availability

Data will be made available on request.
